# A multi-step approach to the treatment of giant scalp congenital hemangiomas: a report of two cases

**DOI:** 10.3389/fsurg.2023.1045285

**Published:** 2023-05-24

**Authors:** Raymond W. Ho, Gabriel Nonnenmacher, Hans Henkes, Christian Vokuhl, Steffan Loff

**Affiliations:** ^1^ Department of Pediatric Surgery, Olgahospital, Klinikum Stuttgart, Stuttgart, Germany; ^2^ Neuroradiological Clinic, Katherinenhospital, Klinikum Stuttgart, Stuttgart, Germany; ^3^ Institute of Pathology, University Hospital Bonn, Bonn, Germany

**Keywords:** transarterial embolization, percutaneous sclerotherapy, congenital hemangioma, propranolol therapy, surgical resection

## Abstract

This is a review of two cases of neonatal giant scalp congenital hemangioma. Both patients were treated with propranolol using a similar multi-step approach that included transarterial embolization of the supplying arteries followed by surgical resection of the lesion. In this report, we discuss the treatments, complications, and clinical outcomes of interventions and surgical procedures.

## Introduction

Congenital hemangiomas (CHs) are rare benign tumors that develop at birth. CHs develop primarily in Caucasian females born prematurely to mothers who underwent chorionic villus sampling (1). These lesions are most frequently located at the head or neck (2) and are classified as either a NICH (non-involuting congenital hemangioma) or a RICH (rapidly involuting congenital hemangioma). RICHs proliferate rapidly in the first few weeks after birth and involute during the first 6–14 months of life (3). Approximately 95% of these lesions will regress spontaneously without further sequelae and thus require no treatment (4). By contrast, NICHs do not regress and may enlarge as the patient ages (5).

Infantile hemangiomas (IH) are on the contrary the most common benign vascular tumors of infancy. They usually involute without significant residua. Indications for intervention include ulceration, prevention of disfigurement, and impairment of function or vital structures (6). There are various options available to treat infantile hemangiomas. These lesions can be treated systemically or topically with lasers, cryotherapy, or surgery. Systemic treatments include propranolol and corticosteroids; timolol and propranolol are commonly used for these indications (7). Although infantile hemangiomas respond well to these medications, they have little to no impact on CHs. Recent reports however, demonstrated the effectiveness of propranolol on large hemangiomas (8).

Giant CHs are often associated with clinical complications, including high output cardiac failure, consumptive coagulopathy, and permanent cosmetic concerns (9). Therefore, these lesions warrant surgical intervention even in cases in which the procedure might generate disfiguring scars (10, 11). Transarterial embolization (TAE) can be performed before surgical excision of the lesion to prevent significant intra-operative bleeding.

Here, we document our experiences with two children who were diagnosed with extracranial giant CHs located in the temporoparietal and retro auricular regions, respectively. Both children were treated initially with propranolol and subsequently with TAE before surgical excision.

## Methods and results

### Institutional case

#### Case 1

A newborn male (birthweight 3,470** **g) was delivered at our hospital via cesarean section during the 38 + 1 gestational week. An antenatal ultrasound examination in the 35th gestational week revealed a lesion on the head accompanied by high output cardiac failure. Elective fetal magnetic resonance imaging (MRI) revealed a large extracranial lesion measuring 6.2** **cm × 2.2** **cm × 5.6** **cm in the temporoparietal region with no apparent impact on brain development.

Physical examination revealed a heterogeneous raised (2.4** **cm) lesion with a circumference of ∼6** **cm. Echocardiography at birth documented right heart failure accompanied by pulmonary hypertension and grade I tricuspid valve regurgitation.

**Figure 1:**
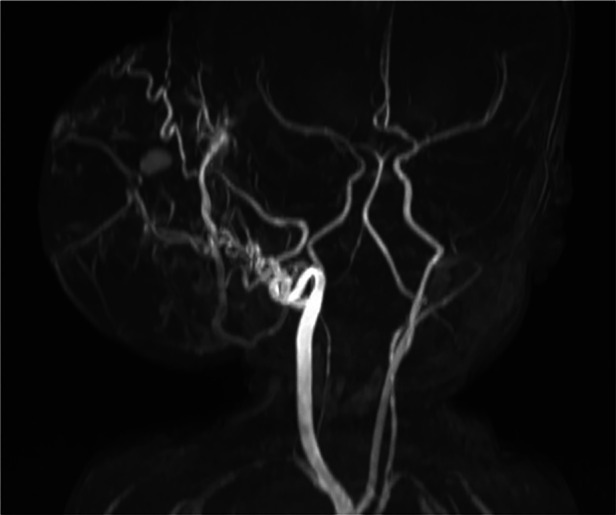
Time-of-flight magnetic resonance angiography revealed a prominent blood supply via the right external carotid artery. Photo courtesy of Thekla von Kalle, Institute for Pediatric Radiology, Olgahospital, Klinikum Stuttgart.

Laboratory results included elevated levels of troponin I (76** **ng/L; normal <59** **ng/L) and N-terminal pro-brain natriuretic peptide (NT-pro-BNP; 6,771 pg/ml; normal <400 pg/ml). Abdominal, spinal, and cranial ultrasound examinations were performed to rule out additional deformities. A repeat MRI study revealed that the highly vascular lesion was supplied primarily by the right external carotid artery (Figure 1). Blood drained from the lesion via the superior sagittal sinus and the left internal jugular vein.

Pediatric cardiology, pediatric surgery, and the interventional neuroradiology departments were consulted for insight into potential treatment approaches. Systemic treatment with propranolol (1** **mg/kg/day) commenced immediately after birth. This treatment was well-tolerated and the patient's vital signs remained stable; the dose was increased to 2** **mg/kg/day on the following day. Echocardiographic studies revealed that signs of early heart failure diminished during the 14 days to follow.

On day 14 after birth, the temporoparietal lesion had grown to 14 cm in circumference and was raised by 2.5 cm. Although the growth of the lesion ceased after this time, the blood vessels supplying the lesion became increasingly prominent; surgical intervention was planned. Successful embolization of the right external carotid feeder artery was performed via insertion of a 4-French introducer sheath into the left femoral artery and a combination of Glubran®2 (GEM s.r.l., Viareggio, Italy) and Lipiodol® (ethiodized oil, Guerbet) in a 1:2–3 ratio was introduced to reduce the risk of intraoperative blood loss. After the procedure, the patient was treated with a continuous intravenous infusion of heparin at 400 IU/kg for 24 h.

Although the sheath in the left groin used for the embolization procedure was removed, the patient developed acute limb ischemia. Doppler ultrasound of the upper regions of the lower left limb revealed thrombosis in both the external iliac and inguinal arteries. The Hematology team recommended a conservative approach (i.e., treatment with a therapeutic dose of low-molecular-weight heparin) to address this complication.

**Figure 2:**
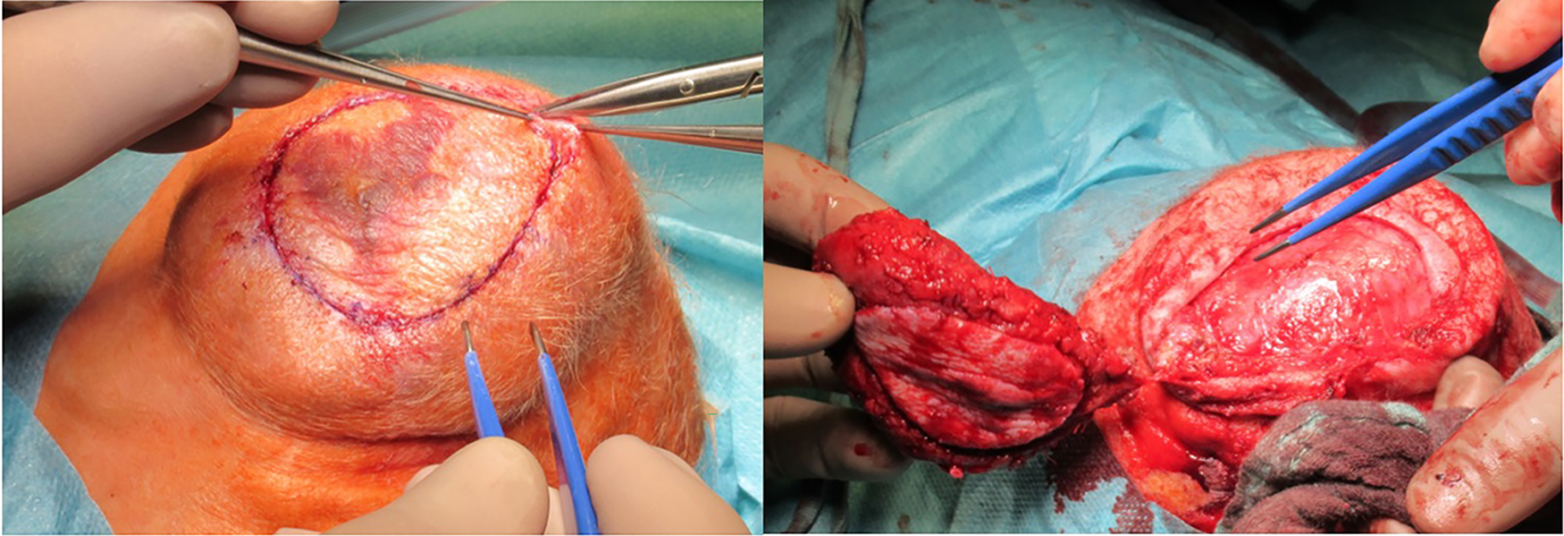
A fusiform incision was followed by outward dissection until the cranium was reached.

As no significant reduction in lesion size was observed, surgical resection of the CH was performed two days after the embolization procedure was completed. The size of the lesion was measured followed by a fusiform incision. To preserve skin for primary closure, the lesion was dissected outwards until the cranium was reached (Figure 2). Careful cauterization was performed to ensure minimal blood loss. Primary wound closure was achieved with continuous sutures, and a pressure wound dressing was applied after completion of the procedure. Observation in the intensive care unit was clinically uneventful.

A Doppler ultrasound examination performed five days later revealed improved vascularity of the popliteal artery due to collateralization of the left iliac artery. Low-molecular-weight heparin was reduced from therapeutic to prophylactic doses after three weeks and continued for six months.

**Figure 3:**
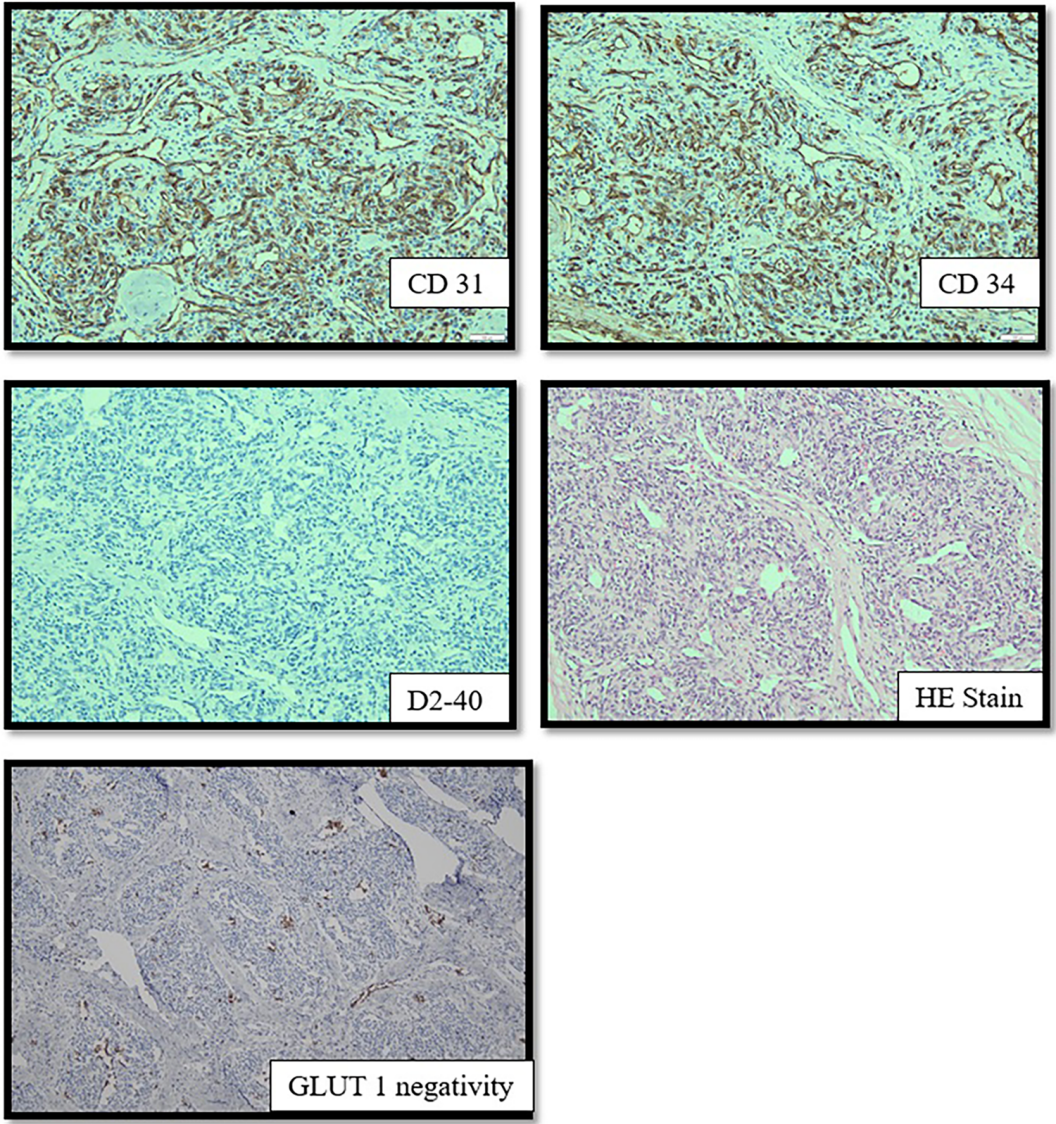
The endothelial cell shows expression of CD31, CD34 and D2-40. GLUT-1 is absent in the immunohistochemistry imaging.

A histopathological evaluation confirmed the diagnosis of CH based on glucose transporter (GLUT)-1 negativity (Figure 3). This analysis also revealed that the resection was incomplete. Propranolol was continued for one week in an effort to treat the CH remnants. A follow-up ultrasound one month after the procedure revealed improved vascularity from the femoral artery and no residual CH. Minor dehiscence of and clear secretions from the surgical incision site were observed at follow-up. These minor complications were treated conservatively with antibiotics and regular dressing changes. Within a month, the wound had closed entirely.

#### Case 2

A newborn female (birthweight 2,810** **g) was transferred to our hospital after a cesarean section during the 37 + 2 gestational week. Non-invasive respiratory support with positive pressure ventilation was required at birth due to poor pulmonary adaptation and APGAR scores of 4/8/10. The patient recovered and began to breathe spontaneously.

A prenatal ultrasound examination revealed an atypically located lesion in the right retro auricular region. An elective postnatal MRI study revealed a high-flow giant extracranial lesion measuring 7.5** **cm × 7** **cm × 4.8** **cm that received blood supply from the right external carotid artery and the middle meningeal artery. Diffuse hemorrhage was observed within the lesion.

Physical examination revealed a large right retro auricular pulsatile mass (approximately the size of a tennis ball) with audible murmurs. The lesion exhibited a firm elastic consistency; dilated veins were prominent on the lesion surface. Echocardiography was performed to rule out cardiac failure.

Laboratory results included elevated D-dimer levels (35,000** **ng/dl) but no other indication of consumptive coagulopathy. Abdominal, spinal, and cranial ultrasounds were performed to rule out additional deformities.

Propranolol therapy (1** **mg/kg/day) was initiated on the second day after birth; the dose was subsequently increased to 2** **mg/kg/day. Propranolol treatment was suspended after a drop in the diastolic blood pressure was observed. The condition of the mass remained unchanged.

**Figure 4:**
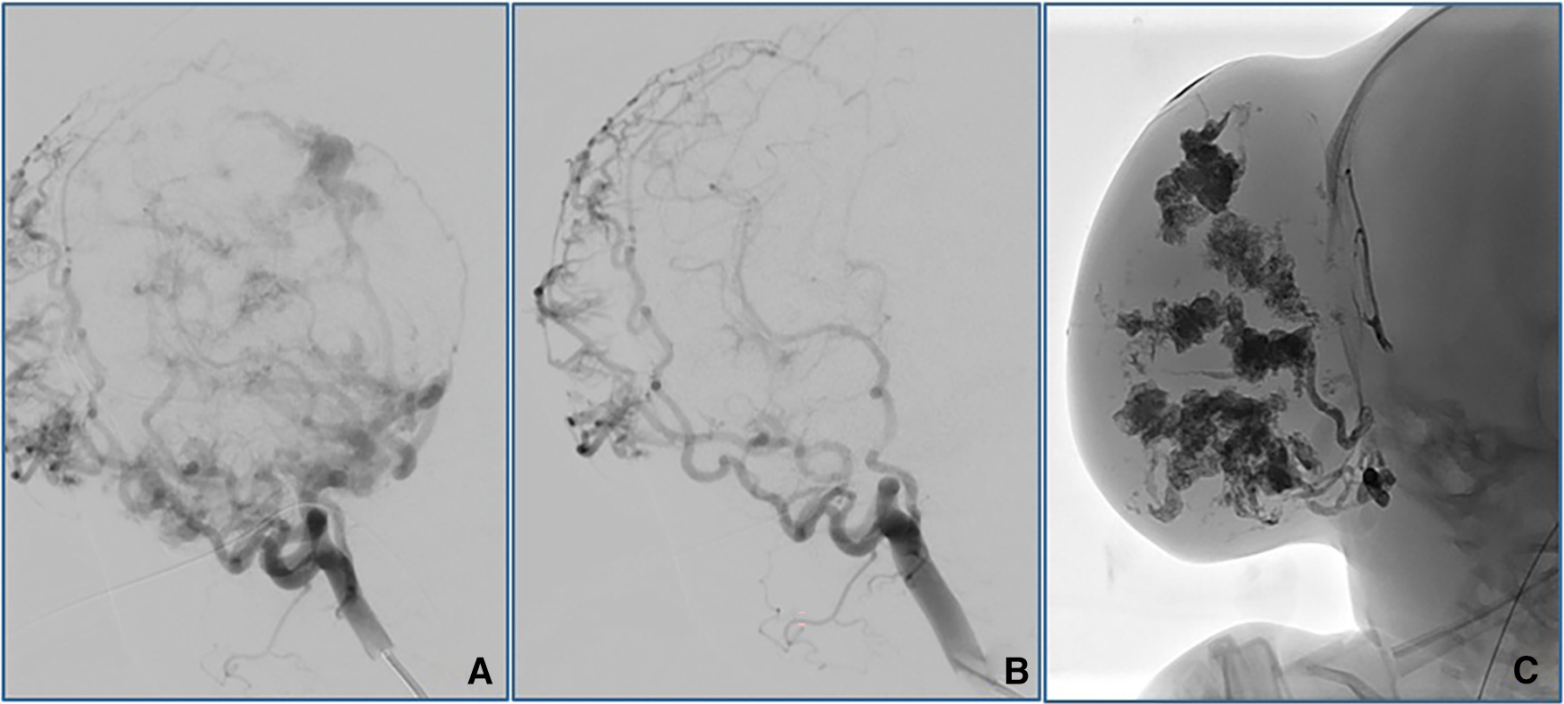
Angiographic outcomes after TAE (**A,B**) and PS (**C**). TAE performed using Magic Glue (Balt)/Lipiodol® (Guerbet) on the right external carotid artery resulted in significant reductions in the arterial blood supply to the lesion. PS with Glubranis®2/Lipiodol® and Magic Glue/Lipiodol® administered locally was performed to eliminate the remaining vessels of the extensive blood supply to this lesion.

To perform TAE, a 4-French introducer sheath was inserted into the patient's right femoral artery. Digital subtraction angiography (DSA) was performed with a 4-F catheter (Tempo4 vertebral; Cordis). A 0.008-inch Marathon^™^ catheter (Medtronic) with a 0.007-inch Hybrid microguidewire (Balt) was used for precise embolization of the branches of the right external carotid artery using a combination of Magic Glue (Balt) and Lipiodol® (Guerbet) in a 1:2–3 ratio. Percutaneous sclerotherapy (PS) with Glubran®2/Lipiodol® and Magic Glue/Lipiodol® in a 1:2 ratio was used to ablate the extensive blood supply to this lesion (Figure 4). Continuous intravenous heparin (400 IU/kg) was provided for the first 12 h after the intervention; the dose was reduced to 200 IU/kg for an additional 12 h.

No signs of limb ischemia were observed upon removal of the sheath from the femoral artery. Prophylactic antibiotic treatment with cefuroxime was administered after serous secretions were found on direct puncture sites on the hemangioma. Although we observed no size regression, the CH developed a softer consistency after these procedures. Ultrasound examination revealed persistent hypervascularization of the lesion after TAE and PS.

**Figure 5:**
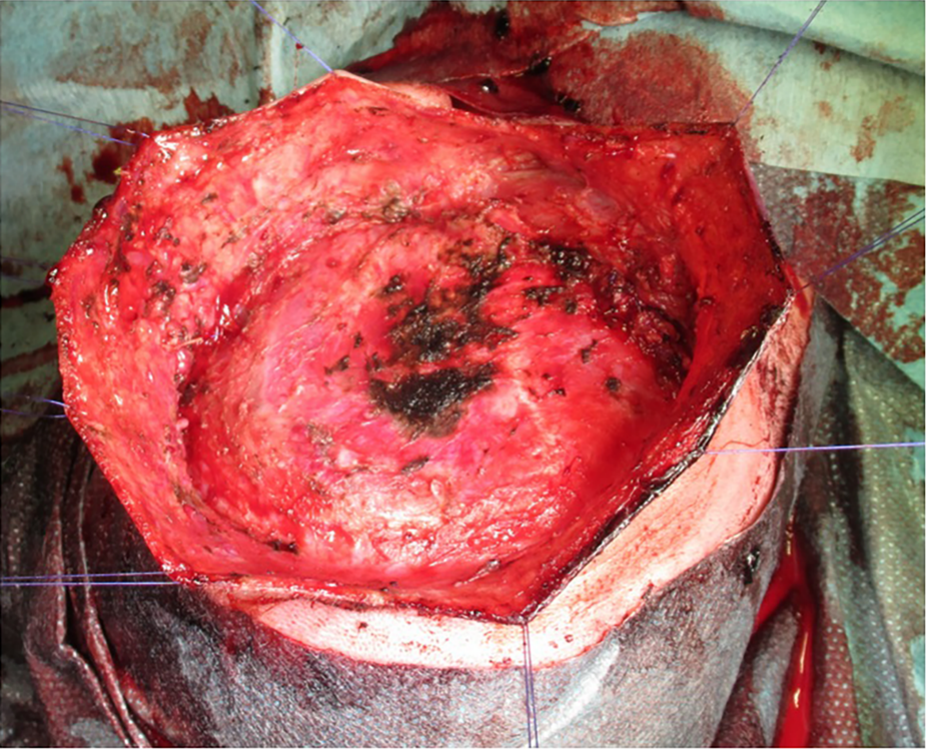
Extensive cauterization of the small vessels supplying the lesion during the surgical intervention. The skin was held by multiple sutures to facilitate easy acess to the lesion.

Surgical resection of the lesion was performed eight days later. Despite the previous embolization procedures, the patient sustained substantial blood loss from the smaller feeder vessels (Figure 5); a blood transfusion was ultimately required. Primary wound closure was achieved. The observation period in the intensive care ward was uneventful.

Histopathological examination revealed overexpression of both CD31 and CD34, confirming its identity as a vascular tumor. The absence of GLUT-1 confirmed the diagnosis of CH which was confirmed as entirely removed. The follow-up evaluation revealed minimal surgical scarring and an excellent cosmetic outcome.

### Literature review

The outcomes and complications of 38 patients from 4 different institutions were tabulated and accessed based on the types of embolization and sclerotizing agents used. Skin necrosis is one of the common complications after transarterial embolization. Demuth (12) reported two patients with skin necrosis, whereby one was major and required a skin graft. This female patient, who had an enlarged right cheek mass, did not initially respond to the embolizing agents (IBCA and Pantopaque) applied to the right maxillary artery. Subsequently, she underwent embolization in the distal branches of the right internal maxillary artery and the right facial artery with double the volume of the same embolizing agents. She suffered extensive skin necrosis thereafter and required multiple excisions and skin grafts. This patient has also developed ipsilateral vision loss after the second embolization attempt.

In Jianhong's (13) case, the two cases of skin necrosis had lesions located in the parotid region. One had TAE (n-BCA and Lipiodol) whilst the other had TAE and BLA. Fortunately, the extent of the necrosis could be treated conservatively using anti-infective measures with regular changes of dressings. Jianhong et al. also reported that another two children who were treated for lesions in the parotid region developed dyspnea on the 3rd day after TAE. Chest x-Rays discovered an iodine deposit, which suggests secondary migration of the embolic agents to the lungs. Both children were treated symptomatically. Five patients who were deemed suitable for surgery after TAE was found intraoperatively that the hemangioma mass has shrunk by 75%. In Jianhong's experience, a single TAE is insufficient, and should surgical intervention be necessary, it should be considered after a month after TAE because significant shrinkage of the hemangioma occurs between the second and the fourth week after TAE.

Patel (14) also demonstrated that at least one treatment with TAE (n-BCA and Lipiodol, ethylene-vinyl alcohol copolymer, microparticles on an inert PVA foam) or PS (Sodium tetradecyl sulfate, ethanol, microfibrillar collagen, thrombin hemostatic matrix and n-BCA) or both is necessary, to prevent progression or recurrence of the lesion within 12 months. Amongst the two cases which required surgical intervention, one patient underwent more than 10 endovascular treatments. Patel's team has highlighted the fact that TAE or PS can reduce the risks of intraoperative bleeding, should surgical intervention be warranted. Treatments using TAE or PS can even omit the need for surgical resection.

Plascencia (15) supported the evidence from other authors. He mentioned that maxillofacial vascular tumors can be treated with TAE and supported that the size of hemangiomas in all 6 cases shrank by 100% within 6 months. PVA was used as an embolizing agent. One patient developed ipsilateral facial hypoesthesia, which may have been attributed by trigeminal ganglion ischemia. Another patient, who was treated with triamcinolone before the treatment with TAE, developed skin necrosis and was advised to undergo skin grafting.

## Discussion

Under the ISSVA classification of vascular tumors, CH falls under benign vascular tumors. It is important to consider other forms of benign vascular tumors which also fall under this category i.e., tufted angioma or Kaposiform hemangioendothelioma. These diagnoses were excluded because of their clinical appearance, as well as mismatching of hematological characteristics such as the absence of profound and sustained thrombocytopenia with profound hypofibrinogenemia and consumptive coagulopathy.

Giant CHs may be associated with heart failure that can be attributed to arteriovenous shunting. Propranolol is a member of the class of drugs known as ß-blockers; the ß2 inhibitory effects of these drugs block the release of vasodilatory transmitters and thus promote vasoconstriction and reduced blood flow to the hemangioma (7). Patient case #1 was treated with a full dose of propranolol (2 mg/kg/day) which resulted in a gradual improvement of the signs of heart failure over time. Alluhaybi et al. (16) suggested that giant CHs require surgical intervention because they typically do not respond to pharmacologic therapy. Results from previous studies revealed mixed results in cases of medically-treated CH including partial, complete, or in some cases, no improvement at all (17–21). As most CHs ultimately involute, the positive outcomes reported may not all be attributed to the effects of medication (22). Our experience with a single case revealed that although propranolol improves cardiac function, it does not promote significant involution of the lesion.

Propranolol also has inotropic and chronotropic effects on the heart and can influence both the heart rate and blood pressure (7). Patient case #2 exhibited a significant decline in diastolic blood pressure in response to propranolol. Administration of the drug was thus discontinued. Both patients underwent surgery to address the aesthetic deformities characteristic of CHs. TAE was performed before excision to reduce the blood supply to the tumor and thus the risk of intra- and postoperative bleeding and to decrease the tumor size. TAE may also reduce the load on the heart and thus improve cardiac function (16). The growth of the lesion can frequently be terminated via TAE or local percutaneous sclerotherapy (PS). Alternative treatments include the usage of Nd: YAG Laser Therapy to reduce the size of the lesion. The cutaneous Nd: YAG treatment has its limitations as it can only penetrate up to 8 mm in depth (23). The tumors presented in both cases had depths >8 mm and was therefore deemed unsuitable. The possibility of endovascular treatment with Nd: YAG using bare-fiber-Technique was excluded as the diameter of the supplying vessels was too large.

Unfortunately, patient case #1 suffered limb ischemia following TAE. Infant blood vessels are short and thin and thus prone to arterial spasm, dissection, and thrombosis. In 1997, Saxena et al. discovered that although intra-arterial heparin was administered during the intervention, thrombosis can occur in about 9% of cases. Fewer attempts at arterial puncture, use of a smaller sheath, maintaining a minimum procedure time, and ensuring that back bleed is present can prevent future occurrences of post-catheterization thrombosis (24). A review article published in 2014 still demonstrated uncertainty on the suitable dosage of heparin to prevent this major complication (25). Similarly, although patient case #2 underwent both TAE and PS, intraoperative blood transfusion was ultimately necessary. This may be because of the extensive branching of the blood vessels associated with the lesion and the challenge involved in attempting to obliterate every source of blood supply. It is also possible that collateralization and recanalization occurred during the week between embolization and the surgical procedure (11, 12).

At this time there are no standard embolic agents recommended for use in TAE or PS. Table 1 lists several studies featuring different agents used in embolization procedures and their respective outcomes. Among them, n-BCA (n-butyl-cyanoacrylate) is commonly used for TAE. At our institution, these procedures are performed using Glubran®2 and Magic Glue (N-hexyl-cyanoacrylate) dissolved in Lipiodol®. Nico L et al. (27) reported that although Magic Glue and n-BCA are distinct chemical structures, they have similar if not identical embolization properties. Glubran®2 is a combined formulation of n-BCA and methacryloxy sulfolane (MS). Although Glubran®2 and standard embolic agents, for example, n-BCA, achieve similar results, Glubran®2 was used more frequently at our institution because it polymerizes via an exothermic reaction at ∼45°C and with a slightly higher polymerization time than n-BCA which reduces the toxicity of the basic monomer (28).

**Table 1:** Literature review summary.

Author	Number of patients	Treatment/Agent used	Complications	Outcomes	Follow up
Demuth et al. (12)	5	TAE Isobutyl cyanoacrylate (IBCA)	• Two patients developed skin necrosis, which required secondary surgery • One patient developed ipsilateral vision loss after surgery	• One patient did not require surgery	• Not reported
Patel et al. (14)	10	TAE 25% n-butyl cyanoacrylate (n-BCA) dissolved in Lipiodol®, ethylene-vinyl alcohol copolymer, microparticles of an inert PVA foam PS Sodium tetradecyl sulfate, 98% ethanol, microfibrillar collagen, thrombin hemostatic matrix, and n-BCA 5 were treated with TAE 2 were treated with PS 3 were treated with combined TAE/PS	• Not mentioned	• Eight patients did not require surgery • One to two required surgical treatment – *One patient had 12 treatments (of which 3 prior endovascular embolization was performed at a peer institution)* – *Another patient had 2 endovascular treatments before surgical intervention* • Five patients only require 1 treatment	• 1–2 months after treatment and at 12 months
Jianhong et al. (13)	17	TAE Coils and an embolic mixture of n-BCA and Lipiodol® PS Bleomycin (BLA) Four were treated with TAE Three had TAE and resection Two underwent TAE and BLA before surgical intervention Eight were treated with TAE and at least one treatment with BLA	• Two patients suffered skin necrosis, which were treated conservatively with antiinfective measures and regular change of dressing • Complications included local pain and dyspnea secondary to migration of the embolic material to the lungs, which occurred in two other patients	• Ten patients required intratumoral injections of bleomycin in lipiodol • Five patients required surgery • None of the patients show signs of recurrence during the follow-up examinations	• Clinical examinations between 5 and 86 months
Plasencia et al. (15)	6 Parotid/cheek IH	TAE Polyvinyl alcohol (PVA)	• One patient suffered left hemifacial hypesthesia	• Surgical intervention for all patients was not required. 100% Tumour shrinkage reported between 2 and 6 months	• Between 2 months up to 2 years
This report	2	TAE 25%–33% Magic Glue (N-hexyl cyanoacrylate) dissolved in Lipiodol® 25%–33% Glubran®2 [n-BCA and methacryloxy sulfolane (MS)] dissolved in Lipiodol® PS 33% Glubran®2 [n-BCA and methacryloxy sulfolane (MS)] dissolved in Lipiodol® and 33% Magic Glue (N-hexyl cyanoacrylate) dissolved in Lipiodol® One was treated with TAE with surgical resection The other was treated with both TAE, PS before surgical resection	• One patient developed limb ischemia secondary to embolism • One patient required an intraoperative blood transfusion	• All patients underwent surgery • One patient had to continue propranolol therapy after histopathological evidence of residual hemangioma. Clinical and ultrasound follow-up examinations showed no signs of recurrence.	• Between 1 months up to 6 months

Patel et al. (14) reported that endovascular embolization procedures and/or direct PS might eliminate the need for surgical resection. The patients typically required one to two treatments to eliminate lesion recurrence or progression within 12 months. Of the 10 NICH patients described in their case series, eight patients did not require surgical intervention. Patel (14) and Jianhong (13) reported that surgical intervention was unnecessary once significant shrinkage of the lesion or complete involution was observed during follow-ups scheduled between 5 and 12 months after the TAE or PS procedure. In our case, we might have delayed the decision to proceed with surgical resection in patient case #1 given that TAE successfully eliminated blood supply to the lesion. This would not have been applicable for patient case #2 because ultrasound examination revealed strong evidence of revascularization of the lesion after TAE and PS. In this situation, we did not anticipate a significant decline in the size of the lesion.

## Conclusion

A giant scalp CH is a rare vascular anomaly that can be accompanied by high output heart failure. Systemic therapy with propranolol is a safe and effective means to treat heart failure in these cases. CHs do not typically respond to this medication. Although both patients in our study underwent treatment with propranolol, its efficacy in this situation remains unclear.

TAE/PS is a secondary approach to CH as it is an effective method that can be used to eliminate the blood supply to these lesions. The decision to proceed with surgical resection after TAE/PS should be decided on a case-by-case basis. Ample time should be allotted to provide the lesion with sufficient opportunity to shrink before considering surgical resection. However, skin necrosis warrants prompt resection. Interdisciplinary cooperation is crucial when designing appropriate methods to treat patients with giant scalp CHs.

## Data Availability

The original contributions presented in the study are included in the article, further inquiries can be directed to the corresponding author.
